# Acupuncture therapy on myofascial pain syndrome: a systematic review and meta-analysis

**DOI:** 10.3389/fneur.2024.1374542

**Published:** 2024-05-03

**Authors:** Jingwen Xiong, Xuancheng Zhou, Xiufang Luo, Xiangjin Gong, Lai Jiang, Qiang Luo, Shengke Zhang, Chenglu Jiang, Tong Pu, Jie Liu, Jun Zhang, Bo Li, Hao Chi

**Affiliations:** ^1^Department of Sports Rehabilitation, Southwest Medical University, Luzhou, China; ^2^Department of Psychiatry, Southwest Medical University, Luzhou, China; ^3^Department of Geriatric, Dazhou Central Hospital, Dazhou, China; ^4^School of Clinical Medicine, Southwest Medical University, Luzhou, China; ^5^College of Acupuncture and Tuina and Rehabilitation, Hunan University of Chinese Medicine, Changsha, China; ^6^Department of General Surgery, Dazhou Central Hospital, Dazhou, China; ^7^Department of General Surgery (Hepatopancreatobiliary Surgery), The Affiliated Hospital of Southwest Medical University, Luzhou, China; ^8^Academician (Expert) Workstation of Sichuan Province, Metabolic Hepatobiliary and Pancreatic Diseases Key Laboratory of Luzhou City, The Affiliated Hospital of Southwest Medical University, Luzhou, China

**Keywords:** acupuncture, myofascial pain syndrome, complementary and alternative therapies, pain, traditional Chinese medicine

## Abstract

**Purpose:**

Traditional Chinese medicine (TCM) therapies, especially acupuncture, have received increasing attention in the field of pain management. This meta-analysis evaluated the effectiveness of acupuncture in the treatment of myofascial pain syndrome.

**Methods:**

A comprehensive search was conducted across a number of databases, including PubMed, Cochrane Library, WOS, CNKI, WANFANG, Sinomed, and VIP. Furthermore, articles of studies published from the inception of these databases until November 22, 2023, were examined. This systematic review and meta-analysis encompassed all randomized controlled trials (RCTs) on acupuncture for myofascial pain syndromes, without language or date restrictions. Based on the mean difference (MD) of symptom change, we critically assessed the outcomes reported in these trials. The quality of evidence was assessed using the Cochrane Risk of Bias Tool. The study is registered with PROSPERO under registration number CRD42023484933.

**Results:**

Our analysis included 10 RCTs in which 852 patients were divided into two groups: an acupuncture group (427) and a control group (425). The results of the study showed that acupuncture was significantly more effective than the control group in treating myofascial pain syndromes, which was reflected in a greater decrease in VAS scores (MD = −1.29, 95% [−1.65, −0.94], *p* < 0.00001). In addition, the improvement in PRI and PPI was more pronounced in the acupuncture group (PRI: MD = −2.04, 95% [−3.76, −0.32], *p* = 0.02) (PPI: MD = −1.03, 95% [−1.26, −0.79], *p* < 0.00001) compared to the control group. These results suggest that acupuncture is effective in reducing myofascial pain. It is necessary to further study the optimal acupoints and treatment time to achieve the best therapeutic effect.

**Systematic review registration:**

https://www.crd.york.ac.uk/prospero/, identifier CRD42023484933.

## Background

The traditional definition of myofascial pain syndrome (MPS) suggests that regional pain originates from hyperirritable spots located within the taut band of skeletal muscle, referred to as myofascial trigger points (MTrPs) (1). Myofascial pain syndrome (MPS) is regarded as one of the most prevalent chronic musculoskeletal pain syndromes. The prevalence of MPS may be as high as 85% in pain clinics (2). Common causes of MPS and dysfunction may include direct or indirect trauma, spinal pathology, exposure to cumulative and repetitive strain, postural dysfunction, and physical disorders (3, 4). The pharmacological management of myofascial pain predominantly involves analgesics and muscle relaxants, with nonsteroidal anti-inflammatory drugs (NSAIDs) being the most frequently prescribed medications. Despite the widespread use of oral NSAIDs, there is a dearth of randomized controlled trials (RCTs) specifically assessing their efficacy for myofascial pain syndrome (MPS). Consequently, there exists a paucity of robust evidence regarding the effectiveness of anti-inflammatory drugs in treating MPS. Moreover, caution should be exercised regarding the prolonged use of oral NSAIDs due to potential gastrointestinal, renal, and antiplatelet adverse effects (5).

Over the past few decades, there has been a notable rise in clinical and scientific attention toward using acupuncture to treat myofascial pain syndrome (MPS). Many clinical studies, especially randomized controlled trials (RCTs), have explored acupuncture’s potential as an intervention for MPS. These studies have consistently demonstrated positive effects of acupuncture in alleviating pain. Several clinical trials and systematic evaluations have indicated that acupuncture can effectively reduce both pain and irritability associated with MPS (6, 7). While the precise mechanism of acupuncture for myofascial pain syndrome (MPS) remains to be fully elucidated, mechanistic studies have concentrated on both peripheral and central aspects. Nevertheless, research indicates that acupuncture can suppress pain transmission by reducing substance P (SP) levels and enhancing the release of endogenous opioids (8, 9). A recent study revealed that acupuncture enhances strength, function, and locomotor activity in a rat model of muscle pain syndrome through its antioxidant effects (10). Additionally, another study demonstrated that acupuncture at trigger points modulated gene expression in muscle tissue, consequently promoting muscle regeneration. Regarding the central aspect, some scholars advocate the notion that acupuncture can activate supraspinal and higher centers engaged in pain processing (11).

Despite the increasing wealth of clinical evidence on the management of myofascial pain in recent years, there is a noticeable absence of more recent meta-analyses focusing on the overall efficacy of acupuncture for this condition. This gap highlights the need for more focused meta-analyses, especially as clinical trials advance. Our proposed meta-analysis aims to fill this void by assessing acupuncture’s effectiveness for myofascial pain using clearly defined outcome measures. The goal of our meta-analysis is to provide valuable insights and information for future clinical treatment strategies, which will be especially helpful for physicians seeking effective approaches to manage myofascial pain.

## Methods

### Search strategy and data mining

For our systematic review and meta-analysis, we searched various literature databases, including PubMed, Cochrane Library, WOS, CNKI, WANFANG, Sinomed, and VIP. The search aimed to identify randomized controlled trials (RCTs) on the effects of acupuncture for myofascial pain syndromes from the inception of each database to November 22, 2023. For the searches, we performed separate searches for acupuncture and myofascial pain, and then combined the results of both searches. We independently conducted a comprehensive review of all relevant published meta-analyses and their reference lists, without imposing any specific limitations on article types. Based on our knowledge, there have been no recent updates on this topic, which supports our claim. The search strategies used in this study are extensively detailed in Supplementary File 1.

### Literature selection

Our inclusion criteria for the retrieved studies were as follows: (1) Diagnosis of “myofascial pain syndrome” based on clear diagnostic (inclusion) criteria (12–14). The patient’s diagnosis was not influenced by other co-morbidities. (2) In these trials, the treatment modality in the experimental group was acupuncture added to the control group. The manipulation and specific acupuncture points used in research are not limited. (3) Any type of control group can be considered as a control group, including traditional western medicine control group, routine care control group and blank control group. (4) Outcomes: Evaluation of the quality of pain management should include at least one of the following scales: 1. Pain Rating Index (PRI). 2. Present Pain Intensity (PPI). 3. Visual Analog Scale (VAS) scores. 4. Efficacy of diagnostic and therapeutic criteria for TCM syndromes. 5. Efficacy of clinical research guidelines for TCM (new medicines) or other meta-analyses referring to extrapolable data on myofascial pain syndromes. 6. The validity of the analysis. Exclusion criteria: patients with one or more other types of pain in addition to myofascial pain syndrome; other interventions such as moxibustion, transcutaneous electrical nerve stimulation, acupoint injections, etc. were used in the study; the paper was only an abstract or review; the study did not have outcome indicators; or the complete literature was not available.

### Data collection

All exclusion and inclusion criteria will be discussed and determined by all researchers prior to the start of the study. At the formal start of the screening phase, each of the two researchers will independently review all study titles and abstracts according to the criteria discussed beforehand, exclude obviously irrelevant literature, and then read the full text of the screened articles. After further screening, the final literature for inclusion was identified, and then the basic information of the articles was extracted along with the data for the set endpoints without knowledge of each other’s review. Finally, the results were cross-checked. When the results of two researchers conflicted, a third researcher stepped in to resolve the disagreement. In the extraction of basic information and data, we mainly recorded the authors of the article and the time of the study, the age of the samples included in the study, the duration of myofascial pain, the number of samples, the measures of the intervention and the control group, the site of pain and the time of application of acupuncture. For the outcome indicators, the values of the primary and secondary outcome indicators were extracted and recorded.

### Quality assessment

We assessed bias in the randomized controlled trials included in the review by means of the Revised Cochrane Risk of Bias Tool (RoB-2) (15). We scored high, medium and low risk based on the risk entries after reading the full text of the included studies, and the specific criteria for each score will refer to Cochrane’s meta-analysis criteria, and then presented the results of our comprehensive bias assessment through the use of Revman 5.4 software, which graphically and clearly depicts the possible bias in these trials, helping us to clearly understand the specific quality and potential risk of the included articles.

### Statistical analysis

Revman software for meta-analysis. Visualization was achieved through forest plots. Statistical analyses were performed using mean difference (MD), and heterogeneity was assessed using the I^2^ index. When the effects showed heterogeneity (I^2^ > 50%), the analysis was performed using a random-effects model; when the data showed homogeneity, the analysis was performed using a fixed-effects model (16).

## Results

### Search results

**Figure 1:**
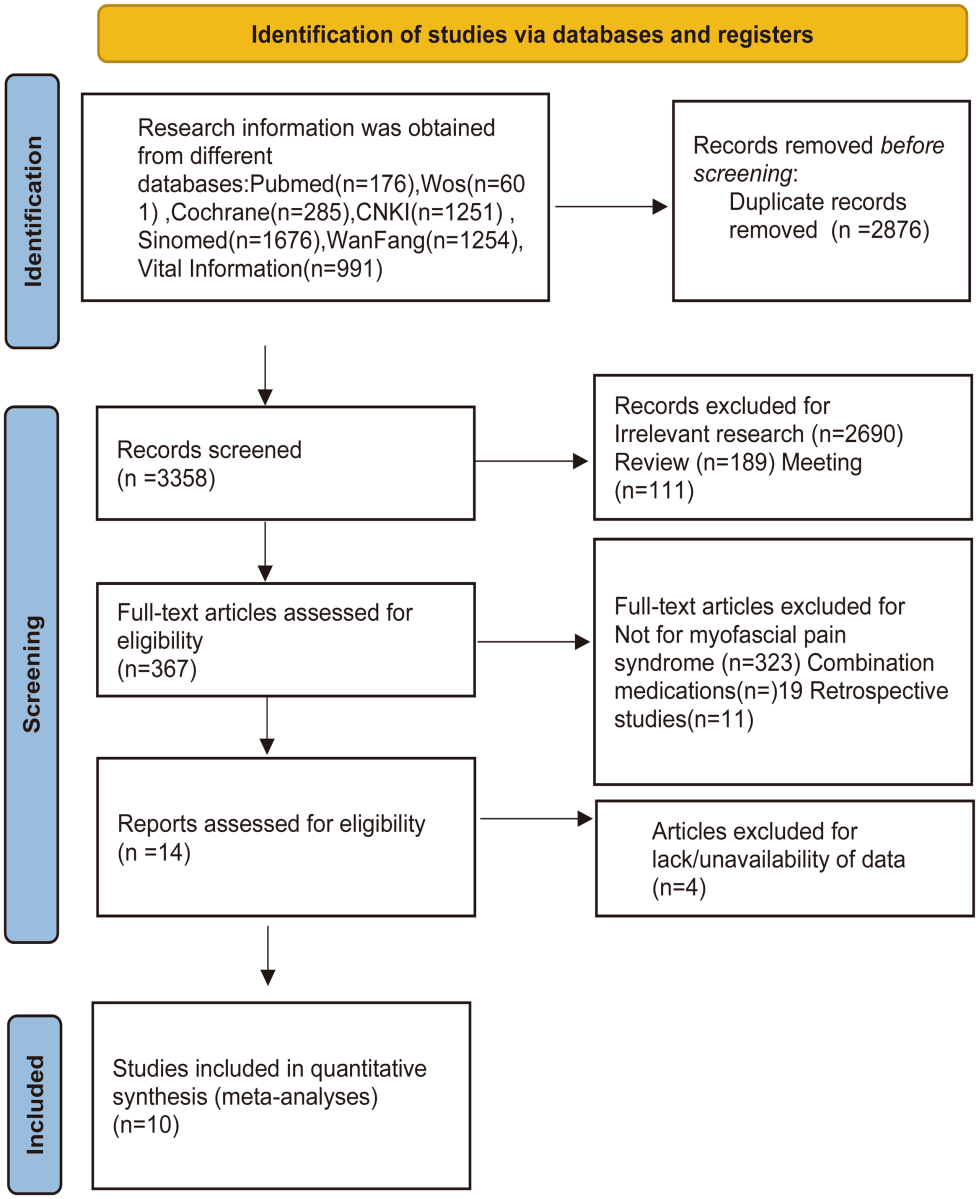
Flow diagram showing the screening and selection process of reports to be included in the meta-analysis.

Initially, a total of 6,234 potential research articles were identified through our search using the designated terms. We then excluded 2,876 duplicate studies using EndNote 20 software. After reviewing titles and abstracts, we identified 2,690 articles that were not relevant to the study and excluded them. In addition, we excluded 300 articles because they were reviews or conference materials. We then thoroughly examined the full text of the remaining 367 articles. Of these, 357 articles were excluded for reasons such as being retrospective studies or not related to acupuncture for myofascial pain symptoms. Ultimately, after careful review, a total of 10 clinical studies met the criteria and were deemed suitable for inclusion in the meta-analysis (17–26) (Figure 1).

### Characteristics of the included studies

A total of 852 patients were included in the 10 trials, including 427 in the acupuncture group and 425 in the control group (17–26). Of the 10 studies, seven groups were treated with direct acupuncture (17, 19, 21–25), and three studies used acupuncture and massage (18, 20, 26). In five studies, the control group used medication for oral treatment (18–21, 26). Several other studies one used rehabilitation (17), one used lidocaine injections (22), one used McKenzie therapy. The remaining two studies control group was treated with herbal medicine (23, 24). For the diagnosis of myofascial pain syndrome, 3 studies used the Pain Science (19, 21, 22). The diagnostic criteria were: the diagnostic criteria were categorized into major and minor criteria, and five major criteria and at least one minor criterion were met to diagnose MPS. Primary criteria: (1) complaints of regional pain; (2) sensory abnormalities in the area of expected distribution of the complaints of pain or trigger point tenderness; (3) palpable tension zone in the affected muscle; (4) intense point tenderness at a point within the tension zone; (5) some degree of restriction of movement during measurement. Secondary criteria: (1) repetition of the complained clinical pain or sensory abnormality at the pressure point; (2) localized twitch response induced by lateral grasping or needle insertion into the trigger point of the band; (3) relief of pain by stretching the muscle or injecting the trigger point. Three studies used the Criteria for Diagnosis and Efficacy of Diseases in Traditional Chinese Medicine (19, 22, 25), and four studies used other criteria such as the Guiding Principles for Clinical Research of New Traditional Chinese Medicines, and Surgical Treatment of Cervical Spine Disease (17, 23, 24, 26). Six of the 10 studies treated neck and shoulder myofascial pain (17–20, 25, 26) and four studies were on low back myofascial pain (21–24). Table 1 shows the main characteristics of the included studies: including the sample sizes of the two groups, the ages of the included patients, the treatments used in the treatment group, the treatments used in the control group, and the duration of myofascial pain syndrome. Regarding the efficacy criteria of the included studies, eight studies assessed the VAS (17–23, 26), four studies assessed the PPI and PRI scores (19, 20, 22, 23), and nine studies assessed the efficacy of the treatments using the treatment criteria of TCM evidence (17–25). Table 2 shows the outcome indicators of the included studies.

**Table 1:** Characteristics of included studies.

Author	Age (years)	Duration of disease (years)	Number of acupuncture group	Number of control group	Acupuncture group	Control group	Location of pain	Frequency of acupuncture	Outcome indicator
Chen et al. (17)	Acupuncture group:57.73 ± 9.81 control group:58.49 ± 10.87	Acupuncture group:0.2 ± 0.08 control group:0.19 ± 0.09	30	30	Control group+acupuncture	Convalescent training	Neck and shoulder	5 times per week for 1 month	Clinical efficacy, VAS
Xiaolu (18)	Acupuncture group:63.32 ± 2.76 control group:63.35 ± 2.73	Acupuncture group:2.11 ± 0.06 control group:2.09 ± 0.05	56	56	Control group+acupuncture and massage	Celecoxib	Neck and shoulder	Every two days for one month	Clinical efficacy, VAS
Xi-liang (19)	Acupuncture group:45.17 ± 5.09 control group:45.87 ± 5.23	Acupuncture group:1.04 ± 0.23 control group:1 ± 0.22	46	45	Control group+acupuncture	Indomethacin + Tizanidine Hydrochloride	Neck and shoulder	Every two days for 14 days	Clinical efficacy, VAS, PPI, PRI
Kang (20)	Acupuncture group:45.72 ± 4.80 control group:45.13 ± 5.04	NA	30	30	Control group+acupuncture and massage	Tizanidine	Neck and shoulder	NA	Clinical efficacy, VAS, PPI, PRI
Hongliang et al. (21)	Acupuncture group:42.96 ± 8.22 control group:43.65 ± 8.62	Acupuncture group:1.94 ± 0.72 control group:1.71 ± 0.73	46	46	Control group+acupuncture	Paracetamol	Lower back	Once a day for 14 days	Clinical efficacy, VAS
Xiongjiang et al. (22)	Acupuncture group:43.96 ± 8.42 control group:44.65 ± 8.72	NA	48	48	Control group+acupuncture	Lidocaine injection	Lower back	Every two days for 20 days	Clinical efficacy, VAS, PPI, PRI
Wang (23)	Acupuncture group:49.58 ± 2.74 control group:49.43 ± 2.89	Acupuncture group:5.98 ± 1.01 control group:5.78 ± 1.09	42	41	Control group+acupuncture	Massage	Lower back	6 times per week for 21 days	Clinical efficacy, VAS, PPI, PRI
Zhijuan etal (24)	Acupuncture group:34.10 ± 9.55 control group:34.12 ± 9.54	Acupuncture group:3.25 ± 1.05 control group:3.24 ± 1.02	49	49	Control group+acupuncture	Hot compress with Chinese medicine	Lower back	1 time per week for 2 weeks	VAS
Yuxia (25)	NA	NA	30	30	Control group+acupuncture	McKenzie therapy	Neck and shoulder	Every two days.	VAS
Hongsheng et al. (26)	Acupuncture group:62.39 ± 5.92 control group:63.51 ± 6.37	Acupuncture group:2.36 ± 5.28 control group:2.41 ± 4.94	50	50	Control group+acupuncture and massage	Celecoxib	Neck and shoulder	Three times a week for one month	Clinical efficacy

**Table 2:** Data on outcome indicators included in the study.

Author	Number of acupuncture group	Number of control group	VAS	Clinical efficacy	PPI	PRI
Chen et al. (17)	30	30	Acupuncture group:1.78 ± 0.81 control group:4.66 ± 1.72	Acupuncture group:20(significant efficiency)28(efficiency) control group:14(significant efficiency)25(efficiency)	NA	NA
Xiaolu (18)	56	56	Acupuncture group:1.44 ± 0.08 control group:1.98 ± 0.21	Acupuncture group:52(significant efficiency)55(efficiency) control group:45(significant efficiency)47(efficiency)	NA	NA
Xi-liang (19)	46	45	Acupuncture group:3.52 ± 0.49 control group:4.79 ± 0.52	Acupuncture group:31(significant efficiency)44(efficiency) control group:18(significant efficiency)39(efficiency)	Acupuncture group:1.16 ± 0.12 control group:1.99 ± 0.16	Acupuncture group:1.57 ± 0.16 control group:2.01 ± 0.18
Kang (20)	30	30	Acupuncture group:1.18 ± 0.32 control group:4.79 ± 0.52	Acupuncture group:18(significant efficiency)28(efficiency) control group:10(significant efficiency)21(efficiency)	Acupuncture group:1.10 ± 0.26 control group:2.45 ± 0.47	Acupuncture group:0.48 ± 0.08 control group:1.09 ± 1.20
Hongliang et al. (21)	46	46	Acupuncture group:2.33 ± 0.22 control group:3.43 ± 0.20	Acupuncture group:31(significant efficiency)40(efficiency) control group:24(significant efficiency)38(efficiency)	NA	NA
Xiongjiang et al. (22)	48	48	Acupuncture group:2.33 ± 0.51 control group:2.67 ± 0.42	Acupuncture group:31(significant efficiency)42(efficiency) control group:29(significant efficiency)43(efficiency)	Acupuncture group:0.83 ± 0.11 control group:1.54 ± 0.24	Acupuncture group:6.48 ± 1.01 control group:9.79 ± 1.08
Wang (23)	42	41	Acupuncture group:2.24 ± 0.80 control group:4.31 ± 0.82	Acupuncture group:31(significant efficiency)41(efficiency) control group:21(significant efficiency)35(efficiency)	Acupuncture group:0.92 ± 0.30 control group:2.21 ± 0.50	Acupuncture group:6.31 ± 1.14 control group:10.14 ± 1.22
Zhijuan et al. (24)	49	49	NA	Acupuncture group:42(significant efficiency)48(efficiency) control group:28(significant efficiency)41(efficiency)	NA	NA
Yuxia (25)	30	30	NA	Acupuncture group:10(significant efficiency)28(efficiency) control group:7(significant efficiency)26(efficiency)	NA	NA
Hongsheng et al. (26)	50	50	Acupuncture group:1.24 ± 0.32 control group:3.12 ± 0.84	NA	NA	NA

### Quality assessment

**Figure 2:**
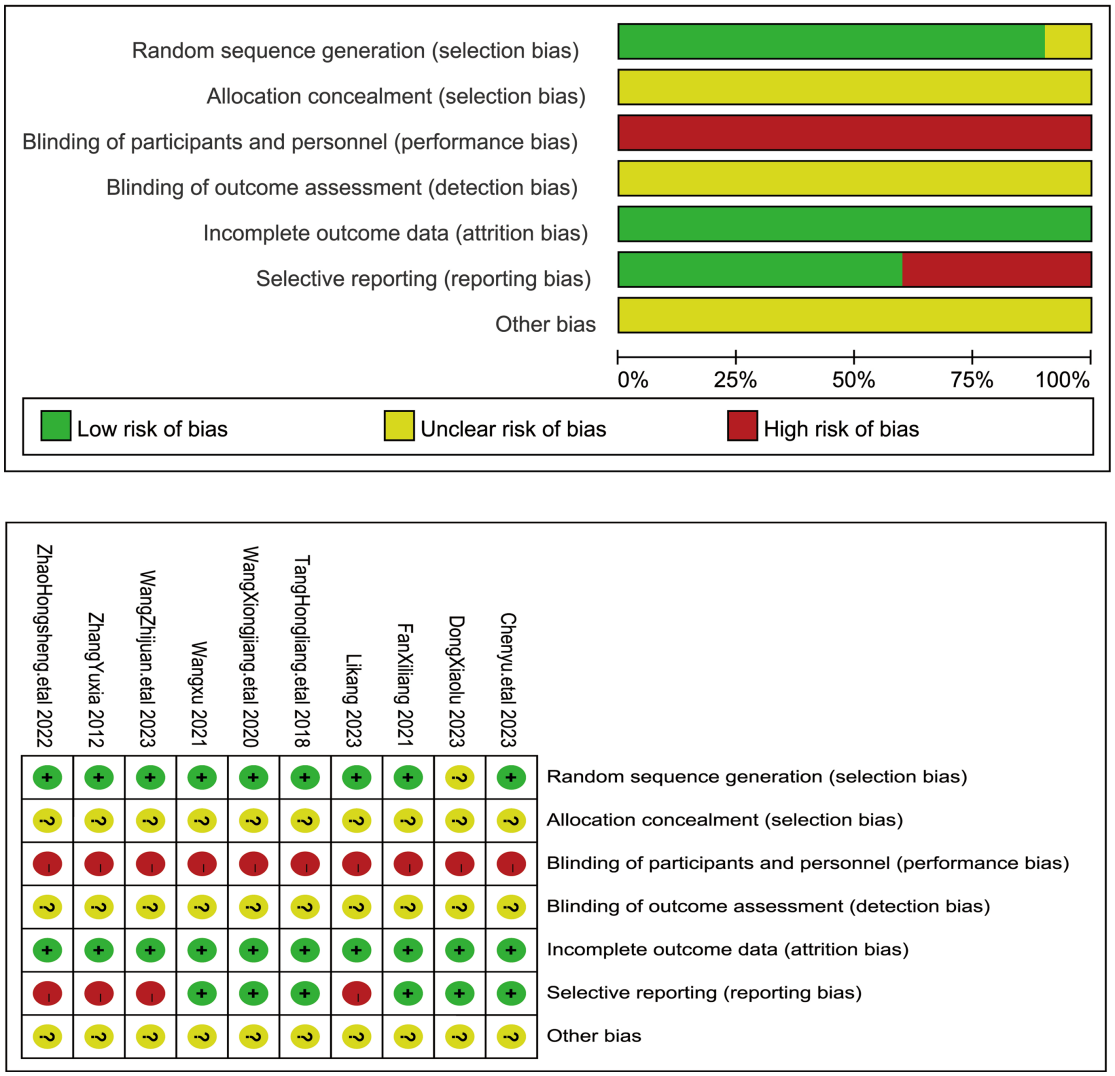
The figure represents the risk of bias assessment for the studies selected in the meta-analysis.

The methodological assessment results are depicted in Figure 2. Out of the 10 studies employing random allocation methods, 9 were appraised as low risk due to the utilization of a randomized table of numbers (17, 19–26), while 1 study was deemed to have an unclear risk of bias due to inadequate information (18). None of these studies provided sufficient detail about the allocation concealment process to warrant a clear risk of bias judgment. Similarly, none of them involved blinding of subjects or administrators due to notable discrepancies in acupuncture treatment utilization between the treatment and control groups. All studies were found to have complete outcome data with a low risk of bias (17–26). Six studies were classified as having a low risk of bias for selective reporting (17–19, 21–23), as they reported all prespecified endpoints. Conversely, four studies were identified as having a high risk of bias for selective reporting due to poor endpoint reporting (20, 24–26). Additionally, insufficient data were available in the 10 studies to assess other risks of bias.

## Results of individual studies

### Main outcome indicators

#### Visual analog scale (VAS)

**Figure 3:**
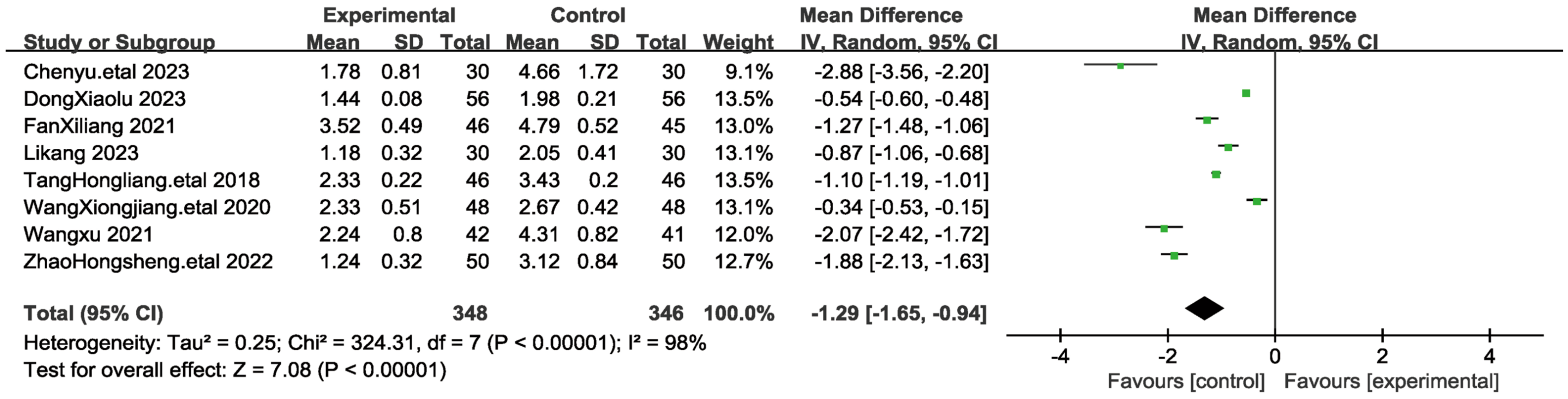
The figure represents a forest plot of the meta-analysis for Visual analog scale (VAS). Each row represents a study and lists the name of the study, the mean systolic blood pressure and standard deviation for the acupuncture and control groups, the sample size, and the mean difference and its 95% confidence interval.

The Visual Analog Scale (VAS) is a commonly used tool for assessing pain intensity. It consists of a horizontal or vertical line, usually 10 centimeters in length, with anchor points at each end representing the extremes of pain intensity (e.g., “no pain” to “worst pain imaginable”). Patients are asked to mark on the line the point that best represents their current level of pain. The distance from the “no pain” end of the line to the patient’s mark is measured and recorded, providing a numerical value that represents the intensity of the pain experienced by the patient. The VAS score can range from 0 to 10 or from 0 to 100, with higher scores indicating greater pain intensity. A total of 8 articles assessed VAS scores in 694 patients, 348 in the acupuncture group and 346 patients in the control group (17–23, 26). Notably, VAS values were significantly lower in patients who underwent acupuncture treatment than in the control group. Due to substantial heterogeneity among these studies (I^2^ = 98%, *p* < 0.00001), we employed a random-effects model. The combined results, as depicted in Figure 3, revealed a statistically significant difference in VAS scores (MD = −1.29, 95% CI [−1.65, −0.94], p < 0.00001). These findings indicate that the acupuncture treatment group exhibited greater improvement in myofascial pain compared to the control group To ensure the stability of our findings, we excluded each study and observed the changes in the combined results after exclusion. We found that the results showed consistency after the exclusion of each study, which demonstrated the stability of our conclusion that the acupuncture group had a significantly lower VAS than the control group after treatment. Therefore, we can state that for myofascial pain, acupuncture treatment is effective in reducing the pain level of patients compared to the control group.

**Figure 4:**
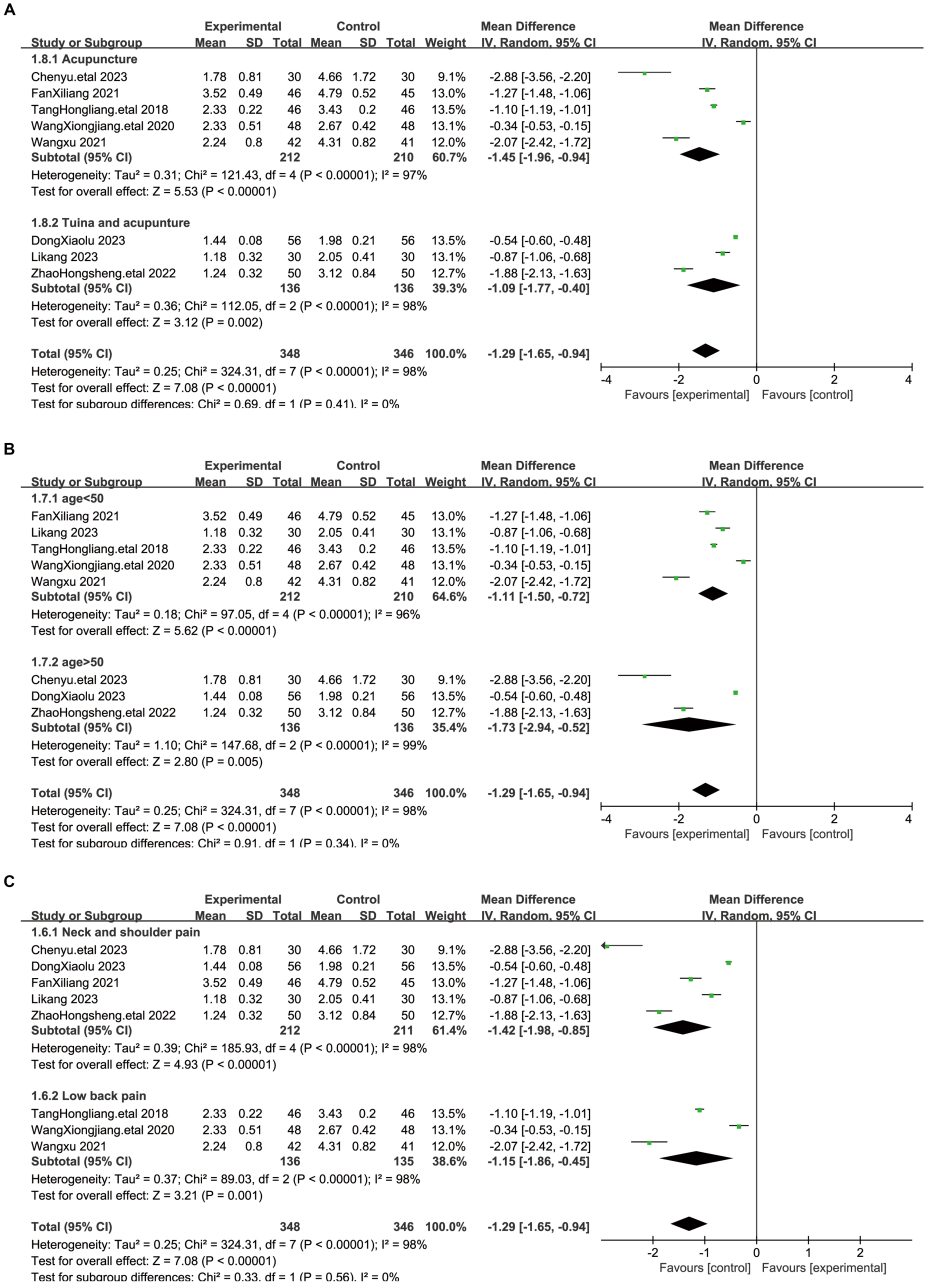
Figure shows a forest plot of subgroup analyses of the visual analog scale (VAS). **(A)** Subgroup analyses regarding different interventions. **(B)** Subgroup analyses of patient age. **(C)** Subgroup analyses of different pain sites.

To ensure the accuracy of the results, we performed subgroup analyses for the different acupuncture interventions in the treatment groups (acupuncture, acupuncture combined with massage) (Figure 4). Acupuncture combined with massage treatments were mainly based on rubbing and pressing on the affected acupoints on the basis of acupuncture, and crossing the hands to squeeze and knead the patient’s painful areas of the tendons in front of and behind the muscles. The results showed that there was a statistical effect of both acupuncture and acupuncture combined with massage on the outcome of myofascial pain (*p* < 0.00001) and there was no heterogeneity between the two groups (I^2^ = 0%). To further investigate the treatment of myofascial pain with acupuncture, we performed a subgroup analysis of patient age and pain site, which showed that differences in patient age and pain site did not affect the results, and there was no heterogeneity between the different subgroups. The results of the subgroup analysis showed the robustness of the results of acupuncture for myofascial pain.

### Secondary outcome indicators

#### Pain rating index (PRI)

**Figure 5:**
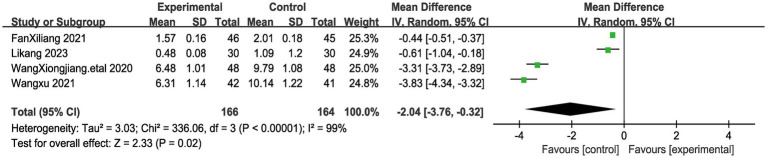
The figure represents a forest plot of the meta-analysis for Pain rating index (PRI).

The Pain Rating Index (PRI) is a component of the McGill Pain Questionnaire, a widely used tool for assessing the quality and intensity of pain. The PRI consists of 78 pain descriptors divided into 20 groups, each representing a different quality of pain (such as throbbing, shooting, stabbing, etc.). Patients are asked to indicate which words best describe their pain, and each selected word is assigned a numerical value based on its rank in the group (e.g., the first word selected is assigned a value of 1, the second word a value of 2, and so on). The PRI score is calculated by summing the numerical values of all selected words, providing a measure of the overall intensity of pain descriptors chosen by the patient. A total of 4 articles evaluated PRI scores in 330 patients, 166 in the acupuncture group and 164 patients in the control group (19, 20, 22, 23). Notably, PRI values were significantly lower in patients who underwent acupuncture treatment than in the control group. Due to the significant heterogeneity between these studies (I^2^ = 99%, *p* < 0.00001), we used a random effects model for the analysis. The results of the combined analysis showed (Figure 5) that there was a significant difference in PRI between the acupuncture group and the control group (MD = −2.04, 95% CI [−3.76, −0.32], *p* = 0.02), suggesting that acupuncture treatment was more effective in improving myofascial pain. To ensure the stability of our findings, we excluded each study and observed the changes in the combined results after exclusion. We found that the results showed consistency after the exclusion of each study, which demonstrated the stability of our conclusion that the acupuncture group had a significantly lower PRI than the control group after treatment. Therefore, we can state that for myofascial pain, acupuncture treatment is effective in reducing the pain level of patients compared to the control group.

#### Present pain intensity (PPI)

**Figure 6:**
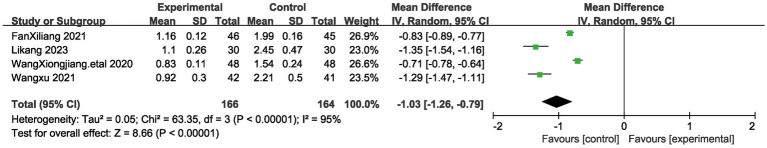
The figure represents a forest plot of the meta-analysis for Present pain intensity (PPI).

Present Pain Intensity (PPI) is a scale used to assess the current level of pain experienced by an individual. It is commonly used in clinical settings and research studies to quantify pain intensity at a specific point in time. The PPI scale typically ranges from 0 to 10, with 0 indicating no pain and 10 indicating the worst possible pain. Patients are asked to rate their current pain level on the PPI scale, providing a subjective measure of their pain intensity. This rating can be used to monitor changes in pain over time, assess the effectiveness of pain management interventions, and guide treatment decisions. A total of 4 articles evaluated PPI scores in 330 patients, 166 in the acupuncture group and 164 patients in the control group (19, 22, 23). Notably, PPI values were significantly lower in patients who underwent acupuncture treatment than in the control group. Due to the significant heterogeneity between these studies (I^2^ = 95%, *p* < 0.00001), we used a random effects model for the analysis. The results of the combined analysis showed (Figure 6) that there was a significant difference in PPI between the acupuncture group and the control group (MD = −1.03, 95% CI [−1.26, −0.79], p < 0.00001), suggesting that acupuncture treatment is more effective in improving myofascial pain. To ensure the stability of our findings, we excluded each study and observed the changes in the combined results after exclusion. We found that the results showed consistency after the exclusion of each study, which demonstrated the stability of our conclusion that the acupuncture group had a significantly lower PPI than the control group after treatment. Therefore, we can state that for myofascial pain, acupuncture treatment is effective in reducing the pain level of patients compared to the control group.

### Diagnostic efficacy criteria for Chinese medicine diseases

**Figure 7:**
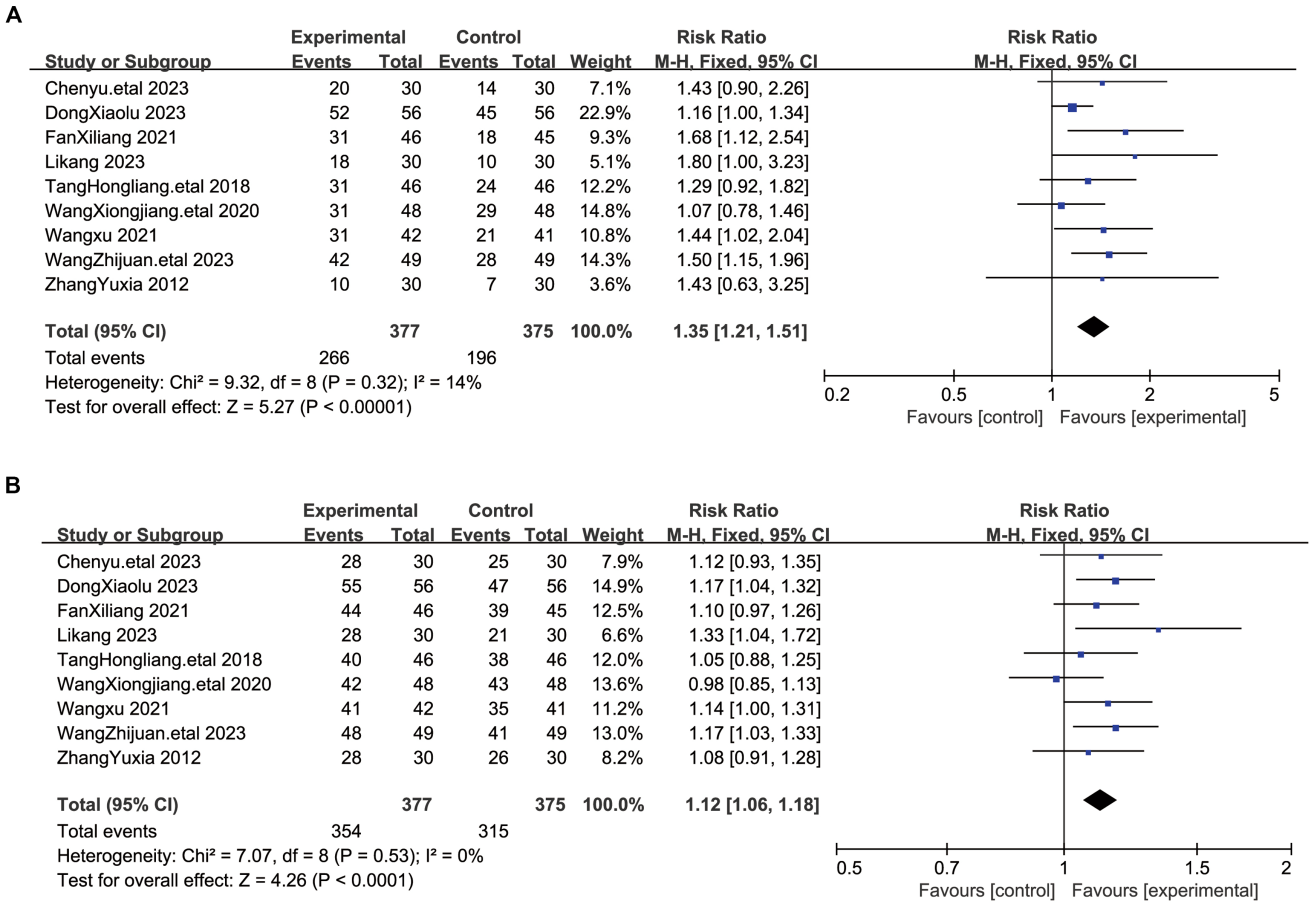
**(A)** The graph shows a forest plot of significant efficacy rates. **(B)** The graph shows a forest plot of total efficacy rates for the treatment of myofascial pain syndromes.

Nine studies evaluated the efficacy of acupuncture treatment involving 752 patients (17–25), and the efficacy was assessed according to the Criteria for Diagnosis and Efficacy of Traditional Chinese Medicine (TCM) Conditions for Myofascial Pain Syndrome. Cure: Symptoms and signs disappear and the patient is able to return to normal work. Significant effect: disappearance of signs and symptoms, no limitation of activity, only pain and discomfort. Effective: improvement of symptoms, reduction of pain, mild limitation of activity; Ineffective: no improvement of symptoms and signs. We combined the cure rate and the significant efficiency rate into the significant efficiency rate. In terms of significant efficacy, we used a fixed-effects model given the low heterogeneity that existed between these studies (I^2^ = 14%, *p* = 0.32). The results indicate (Figure 7A) that the combined treatment shows a significant statistical difference compared to the control group (RR = 1.35, 95% CI [1.21, 1.51], *p* < 0.0001). There was no significant heterogeneity among the studies in terms of overall efficacy (I^2^ = 0%, *p* = 0.52), and we employed a fixed-effect model for analysis. The results show (Figure 7B) that the combined results also exhibit significant statistical significance compared to the control group (RR = 1.12, 95% CI [1.06, 1.18], p < 0.0001). The efficacy rates and overall effectiveness indicate that the acupuncture treatment group is more effective in treating myofascial pain compared to the control group.

## Discussion

Acupuncture stands as one of the commonly utilized alternative therapies. Despite its unclear mechanism of action, the prevailing consensus suggests that acupuncture elicits systemic responses, particularly within the nervous system, through physical stimulation of specific points on the body’s surface. This stimulation regulates bodily functions, ultimately yielding therapeutic effects (27, 28). The publication of various controlled trials has demonstrated acupuncture’s significant efficacy in managing pain syndromes, including acute and chronic low back pain, osteoarthritis of the knee, headaches, myofascial pain, neck pain, and fibromyalgia. Numerous studies have indicated that acupuncture analgesia can be initiated through the stimulation of high-threshold, small-diameter nerves in the muscles (29). These nerves are able to send messages to the spinal cord, which then activates neurons in the spinal cord, brainstem (the gray area around the aqueduct), and hypothalamus (arcuate), which in turn triggers the endogenous opioid mechanism (30–32). A study has shown that pressure-point acupuncture has an analgesic effect and that the intensity of the stimulus may depend on various parameters, such as the procedure, needle size and insertion site. Pressure-point insertion of needles affects sensitized injury receptors, whereas non-pressure-point insertion does not. Pressure pain points are sites where injury receptors (e.g., multimodal receptors) are sensitized by various factors. Moxibustion stimulation of pressure points activates the sensitized multimodal receptors, thereby relieving pain (33). It is also because of its role in myofascial pain that acupuncture is recommended as a treatment option for myofascial pain (34). The results of our meta-analysis showed that acupuncture significantly outperformed the treatment regimen in the control group. This superiority was reflected in lower VAS scores, lower PRI and PPI scores, and higher treatment efficacy in the acupuncture group, and these differences were statistically significant. In order to compare acupuncture therapy and drug efficacy in more depth, we performed a subgroup analysis based on the differences between the treatment protocols of the control group and the acupuncture group, a step taken to explore whether the differences in the treatment groups would affect the reliability of the results, which showed that the different interventions demonstrated good therapeutic effects. Subsequently, subgroup analyses were performed according to the age and pain site of the patients in the different studies. In terms of safety, it is noteworthy that no serious adverse effects were reported in any of the studies, which highlights the fact that acupuncture treatment has a good safety record (35).

While our research findings suggest that the combination of acupuncture and medication is more effective than medication alone for myofascial pain, it is important to acknowledge the limitations of our study. There is significant heterogeneity among the included studies, likely due to differences in the implementation of clinical trials, such as variations in acupuncture point selection, treatment duration and techniques, as well as differences in the types and dosages of medication used in control groups. To comprehensively assess the clinical efficacy of acupuncture in alleviating myofascial pain, future studies should prioritize large-sample, multicenter randomized controlled trials using recognized reliable study designs. We also observed that adverse effects were not systematically studied and documented in the included studies, highlighting the need for future research to verify efficacy. Furthermore, these studies should standardize acupoint selection and treatment methods based on evidence-based principles of traditional Chinese medicine to enhance comparability between treatment studies and facilitate more effective treatments. Additionally, efforts are needed to develop clinical acupuncture treatment protocols that are both efficacious and feasible. This will contribute to the development of evidence-based clinical practice guidelines. Finally, it is important to note that due to the many limitations present in this paper, an updated meta-analysis will be necessary in the future as more clinical trials are conducted. Incorporating higher-quality original studies can provide results with a higher degree of confidence.

## Conclusion

Our study revealed an important finding: the acupuncture group showed significant improvements in VAS scores, PPI and PRI scores, and treatment efficiency compared to treatment with medication alone. This finding provides a solid theoretical basis for the treatment of myofascial pain syndrome with acupuncture. Nevertheless, given the limitations of the existing literature, there is an urgent need for more rigorous and reliable clinical trials to further validate this finding. It may be necessary to conduct an in-depth analysis of different acupoints and intervention times to better explore the factors affecting efficacy.

## Data availability statement

The original contributions presented in the study are included in the article/Supplementary material, further inquiries can be directed to the corresponding authors.

## Author contributions

JX: Writing – original draft, Validation, Formal analysis, Data curation, Conceptualization. XZ: Writing – original draft, Methodology, Formal analysis, Data curation. XL: Writing – original draft, Validation, Project administration, Methodology, Data curation. XG: Writing – original draft, Investigation, Data curation. LJ: Writing – original draft, Investigation, Conceptualization. QL: Writing – original draft, Methodology, Investigation. SZ: Writing – original draft, Project administration, Conceptualization. CJ: Writing – original draft, Project administration, Data curation, Conceptualization. TP: Writing – original draft, Validation, Data curation, Conceptualization. JL: Writing – original draft, Methodology, Funding acquisition, Conceptualization. JZ: Writing – review & editing, Writing – original draft, Methodology, Funding acquisition, Conceptualization. BL: Writing – review & editing, Writing – original draft, Software, Project administration, Methodology, Funding acquisition. HC: Writing – review & editing, Writing – original draft, Visualization, Validation, Software, Conceptualization.
