# Supplementary material for: Acupuncture therapy on myofascial pain syndrome: a systematic review and meta-analysis
Source: Front Neurol. 2024 May 3;15:1374542. doi: 10.3389/fneur.2024.1374542 (PMC11100351; doi:10.3389/fneur.2024.1374542)
Supplement: Supplementary file 1 [file Data_Sheet_1.DOCX]

pubmed:

1. acupuncture[Title/Abstract] OR Electroacupuncture[Title/Abstract] OR electro-acupuncture[Title/Abstract] OR electrosurgical needle[Title/Abstract] OR fire needle[Title/Abstract] OR Fire needles[Title/Abstract] Sort by: Most Recent
2. myofascial pain syndromes[Title/Abstract] OR Myofascial Pain Syndrome[Title/Abstract] OR Pain Syndrome, Myofascial[Title/Abstract] OR Pain Syndromes, Myofascial[Title/Abstract] OR Syndrome, Myofascial Pain[Title/Abstract] OR Syndromes, Myofascial Pain[Title/Abstract] OR Myofascial Trigger Point Pain[Title/Abstract] OR Trigger Point Pain, Myofascial[Title/Abstract] Sort by: Most Recent
3. (acupuncture[Title/Abstract] OR Electroacupuncture[Title/Abstract] OR electro-acupuncture[Title/Abstract] OR electrosurgical needle[Title/Abstract] OR fire needle[Title/Abstract] OR Fire needles[Title/Abstract]) AND (myofascial pain syndromes[Title/Abstract] OR Myofascial Pain Syndrome[Title/Abstract] OR Pain Syndrome, Myofascial[Title/Abstract] OR Pain Syndromes, Myofascial[Title/Abstract] OR Syndrome, Myofascial Pain[Title/Abstract] OR Syndromes, Myofascial Pain[Title/Abstract] OR Myofascial Trigger Point Pain[Title/Abstract] OR Trigger Point Pain, Myofascial[Title/Abstract]) Sort by: Most Recent

Wos

1. (((((((TS=(myofascial pain syndromes)) OR TS=(Myofascial Pain Syndrome)) OR TS=(Pain Syndrome, Myofascial)) OR TS=(Pain Syndromes, Myofascial)) OR TS=(Syndrome, Myofascial Pain)) OR TS=(Syndromes, Myofascial Pain)) OR TS=(Myofascial Trigger Point Pain)) OR TS=(Trigger Point Pain, Myofascial)
2. (((((TS=(acupuncture)) OR TS=(Electroacupuncture)) OR TS=( electro-acupuncture)) OR TS=( electrosurgical needle)) OR TS=(fire needle)) OR TS=(Fire needles)
3. #1 AND #2

Cochrane

1. ‘acupuncture’ OR ‘Electroacupuncture’ OR ‘electro-acupuncture’ OR ‘electrosurgical needle’ OR ‘fire needle’ OR ‘Fire needles’
2. ‘myofascial pain syndromes’ OR ‘Myofascial Pain Syndrome’ OR ‘Pain Syndrome, Myofascial’ OR ‘Pain Syndromes, Myofascial’ OR ‘Syndrome, Myofascial Pain’ OR ‘Syndromes, Myofascial Pain’ OR ‘Myofascial Trigger Point Pain’ OR ‘Trigger Point Pain, Myofascial’

wanfang

（（“针灸”or “针刺”or “火针”or “电针”）and（“肌筋膜疼痛”or “肌筋膜”or “肌筋膜疼痛综合征”or “肌肉骨骼疼痛”or “肌筋膜活动点”or “筋膜疼痛”））

Vip

1. 针灸+针刺+火针+电针

2. 肌筋膜疼痛+肌筋膜+肌筋膜疼痛综合征+肌肉骨骼疼痛+肌筋膜活动点+筋膜疼痛

3. #1 and #2

Cnki

(针灸 + 针刺 + 火针 + 电针) * (肌筋膜疼痛 + 肌筋膜 + 肌筋膜疼痛综合征 + 肌肉骨骼疼痛 + 肌筋膜活动点 + 筋膜疼痛)

Sinomed

（1）"针灸"[常用字段:智能] OR "针刺"[常用字段:智能] OR "火针"[常用字段:智能] OR "电针"[常用字段:智能]

（2）"肌筋膜疼痛"[常用字段:智能] OR "肌筋膜"[常用字段:智能] OR "肌筋膜疼痛综合征"[常用字段:智能] OR "肌肉骨骼疼痛"[常用字段:智能] OR "肌筋膜活动点"[常用字段:智能] OR "筋膜疼痛"[常用字段:智能]

(3) #1 and #2
